# Worrying Affects Associative Fear Learning: A Startle Fear Conditioning Study

**DOI:** 10.1371/journal.pone.0034882

**Published:** 2012-04-13

**Authors:** Femke J. Gazendam, Merel Kindt

**Affiliations:** Department of Clinical Psychology, University of Amsterdam, Amsterdam, The Netherlands; University of New South Wales, Australia

## Abstract

A valuable experimental model for the pathogenesis of anxiety disorders is that they originate from a learned association between an intrinsically non-aversive event (Conditioned Stimulus, CS) and an anticipated disaster (Unconditioned Stimulus, UCS). Most anxiety disorders, however, do not evolve from a traumatic experience. Insights from neuroscience show that memory can be modified post-learning, which may elucidate how pathological fear can develop after relatively mild aversive events. Worrying - a process frequently observed in anxiety disorders - is a potential candidate to strengthen the formation of fear memory after learning. Here we tested in a discriminative fear conditioning procedure whether worry strengthens associative fear memory. Participants were randomly assigned to either a Worry (*n* = 23) or Control condition (*n* = 25). After fear acquisition, the participants in the Worry condition processed six worrisome questions regarding the personal aversive consequences of an electric stimulus (UCS), whereas the Control condition received difficult but neutral questions. Subsequently, extinction, reinstatement and re-extinction of fear were tested. Conditioned responding was measured by fear-potentiated startle (FPS), skin conductance (SCR) and UCS expectancy ratings. Our main results demonstrate that worrying resulted in increased fear responses (FPS) to both the feared stimulus (CS^+^) and the originally safe stimulus (CS^−^), whereas FPS remained unchanged in the Control condition. In addition, worrying impaired both extinction and re-extinction learning of UCS expectancy. The implication of our findings is that they show how worry may contribute to the development of anxiety disorders by affecting associative fear learning.

## Introduction

Emotional memory is considered to lie at the root of anxiety disorders, and originates from a learned association between a previously neutral event (*Conditioned Stimulus or CS*, e.g., stranger) and an anticipated catastrophe (*Unconditioned Stimulus or UCS*, e.g., physical attack). Patients with anxiety disorders feel, think and act as if the feared CS predicts the later occurrence of a catastrophic outcome (UCS). Although Pavlovian fear conditioning serves as a valuable experimental model for studying associative fear memory, it falls short in explaining that most anxiety disorders evolve after relatively mild aversive events rather than traumatic experiences. Insights from neuroscience may shed light on this issue, showing that our memory is continuously updated through an active organization of new information within the context of previous experiences. Hence, processes following fear acquisition may also contribute to the development of pathological fear.

Negative thinking such as worry is a potential candidate for strengthening associative fear memory after fear acquisition. Worry is frequently observed in anxiety disorders [1], [2], and it also predicts anxiety symptoms over time [3]. Worry has been defined as “a chain of thoughts and images, negatively affect-laden and relatively uncontrollable" ([4], p. 10). To account for the negative consequences of worry, specific components have been identified: the repetitive nature, the typical negative valence (e.g., catastrophizing on a real or potential problem), and the abstract level of thinking (e.g., thinking about meanings and implications) [1], [5], [6].

Several mechanisms may explain how worrisome thoughts may strengthen associative fear. First, repeatedly thinking about the fear conditioning experience might both prolong the initial fear reactions as well as strengthen the association between the mental representation of the CS and the UCS, which can lead to increased fear. This can be further explained by recent advances in neuroscience showing that ‘offline’ processes - the processing that continues after (new) learning - may modify the original memory. During the initial memory formation phase (and upon retrieval), memory traces seem to be open to change [7], [8], [9]. The formation of the memory of an event can also be influenced by the emotional reaction following the event [8]. These post-learning processes can strengthen or alter the initial association, potentially resulting in fear enhancement. Second, negative (catastrophic) beliefs (on the perceived threat or about oneself) may increase the threat intensity of the acquired fear memory. This increased threat intensity of the fear associations may not only strengthen subsequent fear responding, but may also strengthen the fear association itself. Previous studies in humans [10] and in rodents [11] have even shown that increased threat intensity enhances fear generalization, a key characteristic of anxiety disorders. Third, worry activates an abstract mode of processing yielding a loss of episodic information [1] and a less concrete representation of the experience [6], [12]. A loss of specificity of the fear acquisition memory may also promote the generalization of fear.

In the present discriminative fear conditioning study, in which one of two neutral pictures (CS1^+^ but not CS2^−^) is paired with an aversive stimulus (i.e. electric stimulus, UCS), we investigated the effects of experimentally induced worry on associative fear memory. For the worry induction we presented the participants with questions regarding their tolerance for and consequences of the anticipated aversive event (electric stimulus, UCS). These questions were based on the three main characteristics of worry (i.e., repetition, negative tone, and abstract style of thinking) [6], [12]. Our study is related to previous work on UCS-inflation that also provides an explanation for the development of anxiety disorders after relatively mild aversive events [13]. UCS inflation refers to the observation that increasing the aversiveness of the UCS following acquisition could enhance the conditioned response to the CS, without additional associative learning [13]. However, the process of UCS-inflation solely involves the aversiveness of the UCS. Note also that the evidence for UCS-inflation is not very robust and exclusively tested for electrodermal responding ([14], [15] and see for critical discussion [16]).

**Figure 1:**
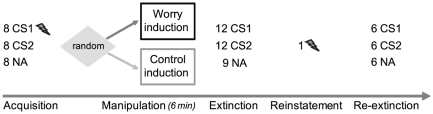
Experimental Design. CS1: stimulus paired with the unconditioned stimulus (UCS; electric stimulus) during acquisition (75% reinforcement rate); CS2: unreinforced stimulus; NA: Noise Alone trials during inter-trial intervals. Flash: electric stimulus.

Here we tested whether a worry induction that immediately follows a fear conditioning procedure would enhance the retention of previously acquired conditioned fear. Conditioned fear responding (CR) was measured as potentiation of the eyeblink startle reflex to a loud noise by electromyography (EMG) of the right orbicularis oculi muscle. Stronger startle responses to the loud noise during the fear-conditioned stimulus (CS1^+^) as compared to the control stimulus (CS2^−^) reflect the fearful state of the participant elicited by the feared CS. The fear potentiated startle (FPS) is considered a reliable and specific index of fear [17], directly connected with and modulated by the amygdala [18]. The cognitive level of conditioning (anticipation of an aversive event) and contingency awareness of the fear association were captured by online UCS expectancy ratings during each CS presentation. We obtained skin conductance responses (SCR) as a more objective measure of UCS anticipation [19], [20] - given that SCR is less sensitive to possible demand effects of our worry manipulation than subjective UCS expectancy ratings. After differential fear conditioning, participants were assigned to either the Worry induction or the Control condition. We tested the effect of worry on the formation of associative fear memory after the worry manipulation. Furthermore, we included an extinction and reinstatement procedure (see Figure 1) to investigate whether worrying would impair the ‘unlearning’ of the fear-conditioned behavior and enhance the recovery of fear. Specifically, we tested whether the worry manipulation would: 1) enhance fear expression at immediate testing (i.e., stronger differential (CS^+^/CS^−^) responding), 2) promote fear generalization to the safe stimulus (i.e., enhanced responding to the CS^−^), 3) impair extinction learning (i.e., diminished reduction in differential responding, 4) generate stronger return of fear (i.e., stronger differential responding at reinstatement testing), and 5) impair re-extinction learning - relative to the control manipulation. Predictions were equal for the three conditioned response measures, FPS, SCR and UCS expectancy, except that we did not predict an effect of worry on UCS expectancy at immediate testing as this measure generally reaches maximum CS^+^/CS^−^ differentiation following an acquisition procedure.

## Results

### Descriptive statistics

No differences in age, sex, trait or state anxiety were found between the Worry and Control condition (all *t*s<1.2) (see Table 1).

**Table 1:** Participant characteristics and UCS characteristics.

	Worry (*n* = 23)	Control (*n* = 25)	T-test	
	*M (SD*)	*M (SD)*	*t* (46)	*p*
*Demographic*s				
Age	22.28 (4.90)	22.12 (2.71)	.14	.886
Sex	19 females	18 females	1.17	.248
*Anxiety*				
State Anxiety	39.17 (9.37)	36.84 (9.23)	.87	.389
Trait Anxiety	40.00 (8.15)	37.20 (9.08)	1.12	.268
*UCS Characteristics*				
Selected UCS intensity	10.13 (5.09)	14.00 (1.41)	1.54	.134
Experienced intensity of UCS (0 = light to 10 = unbearable)	4.38 (1.69)	4.60 (1.53)	.47	.642
UCS unpleasantness, annoyance (0 = not unpleasant to 10 = very unpleasant)	6.14 (1.36)	6.44 (1.85)	.64	.527
Frightened by the UCS (0 = not at all to 10 = very strong)	6.23 (1.76)	6.64 (1.49)	.87	.388

Means and SDs of the Demographics, State and Trait Anxiety [41] and UCS Characteristics of the Worry and Control condition separately. All values represent raw, nonstandardized scores.

#### Manipulation check (see Appendix S2)

Results on participants' compliance with the instructions are presented in Appendix S2. We compared the included and excluded participants on several participant characteristics. Within the Worry condition, included participants (*n* = 23) did not differ on trait anxiety, *t*<1.2, but did show higher state anxiety compared with excluded participants (*n* = 8), *t*(29) = 2.26, *p* = .032, indicating that low state anxious individuals apparently had more difficulty to engage in worrying. Within the Control condition, no differences on participant characteristics were observed, all *t*s<1.

#### UCS characteristics (see Table 1)

Self-calibrated UCS (electric stimulus) intensities ranged from 4 to 55 mA with a mean of 12.15 mA (*SD* = 9.08). After the experiment, the electric stimulus was rated as moderately to strongly aversive on all dimensions. No differences between conditions were observed for selected UCS intensity or in subjective experience of UCS characteristics (all *t*s<1.6).

### Fear-potentiated startle (Figure 2)

**Figure 2:**
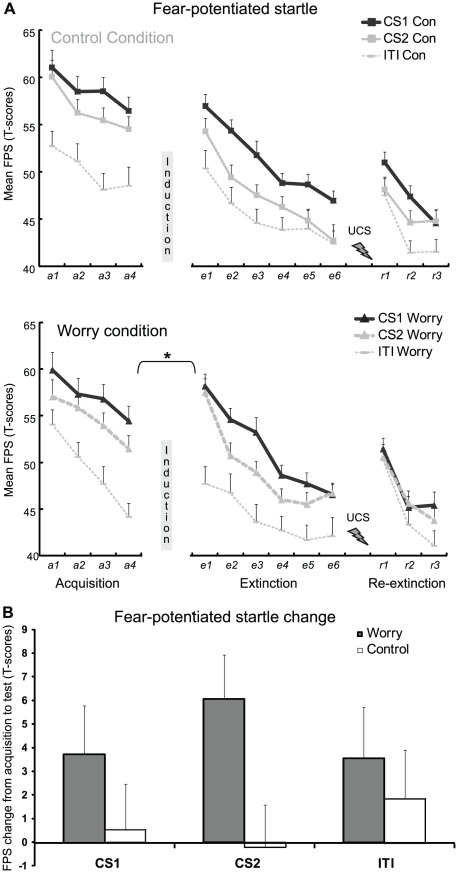
Worry manipulation after acquisition increases fear at test. A. Mean fear-potentiated startle (FPS; standardized T-scores) to the feared stimulus (CS1), safe stimulus (CS2) and during inter-trial intervals (ITI) during acquisition, extinction, reinstatement test and re-extinction for the Control condition (Con) and Worry condition separately. B. Mean change in startle responding to the feared stimulus (CS1), safe stimulus (CS2) and during inter-trial intervals (ITI) from the end of acquisition to test (start of extinction) for the Worry and Control condition (Con). Induction: worry or control manipulation; UCS with Flash: electric stimulus administered during reinstatement; Error bars reflect SEM.

#### Acquisition

The ANOVA did not reveal the CS-Type×Trial interaction from the start to the end of acquisition, *F*<1. Analyses over all acquisition trials demonstrated a significant main effect of CS-Type, *F*(1,40) = 15.98, *p*<.0001, *η*_p_^2^ = .29, indicating higher mean FPS to the feared (CS1^+^) than to the safe (CS2^−^) stimulus. Further, whereas no differential (CS1^+^ vs. CS2^−^) FPS was observed at the start of acquisition, *F*<1.2, we observed a significantly stronger FPS to CS1^+^ than to CS2^−^ at the end of acquisition, *F*(1,40) = 5.00, *p* = .031, *η*_p_^2^ = .11. This indicates that the difference between CS1^+^ and CS2^−^ is the result of acquisition, and is not due to an initial difference in responding at the start of acquisition. Most importantly, the rate of fear conditioning did not differ between conditions, *F*s<1.

#### Post-Manipulation Test

The ANOVA revealed no CS-type×Trial×Condition interaction, *F*<1.3, but a significant Trial×Condition interaction effect emerged from the end of acquisition to the first test trial following the manipulation, *F*(1,40) = 4.14, *p* = .048, *η*_p_^2^ = .09. Post-hoc analyses revealed an increase in FPS responding to both the feared (CS1) and safe stimulus (CS2) in the Worry condition, as illustrated by a significant main effect of Trial, *F*(1,19) = 13.01, *p* = .002, *η*_p_^2^ = .41, while FPS to both CSs remained unchanged in the Control condition, *F*<0.1 (see Figure 2). In addition, while differential (CS1>CS2) startle responding was no longer observed in the Worry condition at test, *F*(1,19)<1, differential startle acquisition was retained in the Control condition, as shown by a trend effect of CS-Type, *F*(1,21) = 3.21, *p* = .083, *η*_p_^2^ = .14. To test whether the foregoing effect could be attributed to the worry manipulation and did not result from a pre-existing acquisition difference or a general (baseline) increase in startle responses, additional analyses were performed. First, analyses confirmed that conditions did neither differ in differential FPS on the last acquisition trial, *F*<1, nor in mean FPS (to both CSs) during acquisition, *F*<2.1. Second, the effect can also not be explained by a general increase in startle responses, as indicated by the absence of a Condition×Trial interaction on NA trials during the intertrial intervals (ITI) from acquisition to test, *F*<1. In sum, the Worry manipulation resulted in increased FPS to both the feared (CS+) and safe stimulus (CS−) at test, while FPS remained stable in the Control condition.

#### Extinction

The ANOVA did not yield a CS-type×Trial interaction from the start to the end of extinction, *F*<1, but a significant linear main effect of Trial was observed, *F*(1,40) = 99.12, *p*<.0001, *η*_p_^2^ = .71, indicating a decline in FPS to both CSs. Since startle responding was also elevated to the CS2^−^ at the start of extinction, a general decrease in startle responding was shown. Further, analyses showed no CS-Type×Trial×Condition interaction, *F*<1, but did reveal a CS-Type×Condition interaction, *F*(1,40) = 4.46, *p* = .041, *η*_p_^2^ = .10. First, this indicates that the conditions did not differ in differential extinction learning, but they differed in overall differential startle response. Follow-up analyses showed that this effect was due to elevated startle to the CS2 in the Worry condition, *F*(1,40) = 9.01, *p* = .005, *η*_p_^2^ = .18, and not CS1, *F*<.2.

#### Reinstatement

The unpredictable UCS (i.e., reinstatement testing) generated an increase in FPS to both CS1 and CS2 from the end of extinction to the start of re-extinction, *F*(1,39) = 21.74, *p*<.0001, *η*_p_^2^ = .36. No interactions with Condition were observed, *F*s<1.7. These results indicate that the effect of Worry induction did not extend to reinstatement testing.

#### Relearning of extinction

The subsequent analysis on re-extinction neither revealed condition differences, *F*s<1.8. Analyses showed only a significant main effect of Trial, *F*(1,39); = 32.94, *p*<.0001, *η*_p_^2^ = .46, indicating a general decrease in FPS.

### UCS expectancy (Figure 3)

**Figure 3.Worry:**
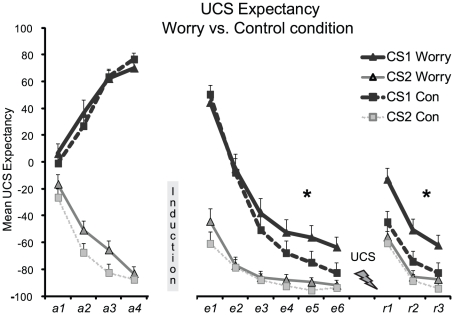
Worry manipulation after acquisition impairs extinction and re-extinction at the cognitive level of conditioned responding. Mean UCS expectancy ratings for the feared stimulus (CS1) and safe stimulus (CS2) during acquisition, extinction, reinstatement test and re-extinction for the Worry and Control condition (Con). Induction: worry or control manipulation; UCS with Flash: electric stimulus administered during reinstatement; Error bars reflect SEM.

#### Acquisition

Successful contingency learning was shown by a significant CS-type×Trial interaction, *F*(1,46) = 365.29, *p*<.0001, *η*_p_^2^ = .89, indicating CS1^+^ had become a meaningful predictor for the UCS and CS2^−^ for the non-occurrence of the UCS. Acquisition patterns did not differ between conditions, *F*<1.4.

#### Post-Manipulation Test

Following the inductions, participants showed a clear decrement in differential UCS expectancy (CS1 vs. CS2) as was indicated by a significant CS-type×Trial interaction, *F*(1,46) = 36.13, *p*<.0001, *η*_p_^2^ = .44. We observed no difference between conditions, *F*<2.1.

#### Extinction

The ANOVA revealed the expected CS-type×Trial×Condition interaction from the start to the end of extinction, *F*(1,46) = 4.68, *p* = .036, *η*_p_^2^ = .09, indicating reduced extinction learning for the Worry condition compared to the Control condition (Figure 3). First, the extinction procedure yielded a significant decrease in differential UCS expectancy inboth conditions (CS-Type×Trial interaction; Worry: *F*(1,23) = 25.18, *p*<.0001, *η*_p_^2^ = .53; Control: *F*(1,24) = 52.07, *p*<.0001, *η*_p_^2^ = .69).

Next, FDR corrected comparisons showed no differences between conditions at the start of extinction (*F*s<1.7) whereas at the end of extinction, the Worry condition continued to show differential UCS expectancy, *F*(1,22) = 12.30, *p* = .002, *η*_p_^2^ = .36, whereas Control participants did not (*F*<4.1). This effect could not be attributed to differences in expectancies for CS2 (all *F*s<1.7), but to elevated UCS expectancy for the CS1 (trend CS1×Condition interaction; *F*(1,46) = 3.24, *p* = .078, *η*_p_^2^ = .07), suggesting that participants in the Worry condition were still not certain about the non-occurrence of the UCS. Together, our results suggest that the worry induction reduced extinction learning.

#### Reinstatement

For both conditions, the reminder UCS produced an increase in UCS expectancy for both the feared and safe stimulus, from the end of extinction to the first test trials, *F*(1,45) = 71.40, *p*<.0001, *η*_p_^2^ = .61. Further, the analyses yielded no CS-Type×Trial×Condition interaction, but a CS-Type×Condition interaction emerged, *F*(1,45) = 4.23, *p* = .045, *η*_p_^2^ = .09, indicating that the Worry condition rated more differential (CS1>CS2) UCS expectancy overall. Subsequent pairwise comparisons revealed that conditions did not significantly differ in differential UCS expectancy at the end of extinction (*F*s<2.2), but that participants in the Worry condition showed stronger differential reinstatement of UCS expectancy than Control participants (CS-Type×Condition interaction; *F*(1,45) = 3.95, *p* = .053, *η*_p_^2^ = .08). Together, our results suggest that the worry manipulation enhanced return of shock expectancy.

#### Re-extinction

In both conditions, re-extinction of UCS expectancy was indicated by a significant effect of Trial, *F*(1,45) = 71.31, *p*<.0001, *η*_p_^2^ = .61, without a CS-Type×Trial interaction. This general decrease may be due to the finding that UCS expectancy to CS2 was also elevated at the first reinstatement test trials. The conditions differed in their mean differential (CS1 vs. CS2) UCS expectancy, as shown by a near significant CS-type×Condition interaction, *F*(1,45) = 3.80, *p* = .053, *η*_p_^2^ = .08, without a CS-Type×Trial×Condition interaction, *F*<1 (Figure 3). Subsequent comparisons again showed that the Worry condition rated significantly stronger differential UCS expectancy than the Control condition at the end of re-extinction (trend CS-Type×Condition interaction; *F*(1,45) = 3.64, *p* = .069, *η*_p_^2^ = .07). To further explore the patterns of re-extinction learning, pairwise comparisons (FDR corrected) showed that the Worry condition continuously rated significantly higher UCS expectancy for the feared (CS1+) than for the safe stimulus (CS2−) at every re-extinction trial, all *p*s<.0005, while the Control condition did not show differential UCS expectancy at any trial, all *p*s>.05. Together, our results indicate that the worry induction resulted in impaired extinction of shock expectancies, enhanced return of shock expectancy and reduced re-extinction learning.

## Discussion

The present study provides experimental support for the prediction that worrying about feared outcomes can affect associative fear learning. Our results demonstrate that worrying after fear acquisition can enhance subsequent conditioned fear to both the conditioned and safe stimulus and can impair extinction at the cognitive level of conditioned responding. Specifically, several minutes of rehearsing catastrophic statements on the personal consequences of a noxious event (i.e., the electric stimulus) resulted in enhanced fear-potentiated startle (FPS) to the originally feared stimulus (CS^+^) and a generalization of fear to the safe stimulus (CS^−^). In contrast, fear responses remained unchanged in the control condition. Note that the elevated conditioned fear response cannot merely be attributed to a general arousing effect, as worry did not enhance startle responding to the context (during the inter-trial intervals). Our worry induction elevated the immediate startle fear responses but the effect was not extended to later phases of testing (i.e., extinction, reinstatement and re-extinction). Further, as expected, the worry manipulation impaired extinction learning of UCS expectancy, generated a stronger return of differential UCS expectancy (reinstatement testing) that persisted to impair *re*-extinction learning.

Our finding that post-acquisition worrying affects the formation of fear memory can be explained by the literature on memory consolidation, which shows that a memory trace can be changed after its original acquisition by either neurobiological or behavioral manipulations [7], [8], [21]. Note that we did not observe evidence for the alternative account of UCS inflation [13] as our retrospective evaluation of the UCS did not differ between conditions. Interestingly, the current finding that worrying enhanced subsequent physiological fear responding seems at first difficult to reconcile with the original claim that worry is associated with a suppression of emotional responding [22]. However, a recent review and re-reanalysis of the existing experimental data on the effects of worry [23] revealed that the majority of experimental studies actually show that worry facilitates and maintains a sustained negative emotional state during the worry process itself [24]. In fact, re-analyzing previous studies in which a dampening effect of worry on subsequent physiological reactivity were found revealed that these effects are confounded by baseline differences [23]. More specifically, if one already experiences negative arousal during worry, and worry is used as the comparison baseline, it appears that prior worry only prevents a further increase in emotional responding [25], [26]. When a pre-worry resting baseline is used as the comparison point, there is no evidence for a muting effect of worry on reactivity to fear stimuli ([26]–[30] and see for review and discussion [23]). Even though our data do not allow drawing conclusions on the effects of worry during the process itself - as we did not measure reactivity during the worry manipulation - they are consistent with the propositions that worry 1) facilitates negative emotional reactivity [23] and 2) prolongs the cognitive representation of the stressor and its concomitant negative emotional state [24].

Although the effect of worrying was immediately expressed at the more implicit, psychophysiological level of fear (fear-potentiated startle), we observed a delayed effect at the more explicit, cognitive level of conditioning (UCS expectancies) (i.e., impaired (re−)extinction learning). One explanation for the absence of sustained effects on startle responding may be that the extinction training - which is a robust manipulation in itself - overruled the effect of the current worry induction. Our manipulation of ‘state worry’ by a *verbal, cognitive* task may have been too subtle to compete with the extinction manipulation to affect physiological responding. On the other hand, the present worry manipulation did impair extinction learning at the cognitive level of conditioning (UCS expectancy) and this effect persisted throughout re-extinction learning. It should be noted that this elevated differential UCS expectancy during reinstatement and re-extinction can be a consequence of incomplete extinction. The present findings of impaired extinction are in line with the recent proposition that worry reduces the capacity for emotional learning. Worry may affect the processing of emotional information in such a way that it interferes with learning from experience [23]. This deficient emotional information processing may contribute to the maintenance of anxious meanings attributed to stimuli (e.g., feared CS) [23].

Our present findings extend previous studies by demonstrating for the first time an effect of experimentally induced worry on *conditioned fear responding*. Furthermore, our worry manipulation about the personal negative consequences of the noxious event (i.e., electric stimulus) also produced a fear response to the safe stimulus. This effect may be interpreted as generalized fear responding and is in line with other studies showing that generalization is dependent on fear intensity (in humans: [10], in rodents: [11]). Stronger generalization of conditioned fear to safe stimuli has also been observed – without any manipulation - in both individuals at risk for anxiety and patients with anxiety disorders (e.g., Gazendam, Kamphuis & Kindt, Unpublished Data, [30]). Fear generalization may be interpreted from a functional perspective. Upon a fearful experience, we are automatically in search for predictors of the event in order to prepare for future encounters [31]. If the expected outcome is perceived as more catastrophic, one may rely on a more generalized class of predictors in order to minimize the risk of ‘missing’ the catastrophe.

The current study was limited in that the effects of worrying were only observed for the startle reflex and UCS-expectancies, but not for electrodermal activity (SCR). Also, the main effect of worry on the fear-potentiated startle was short-lived, that is, it did not extend to phases beyond the first test phase. It should be noted that we only tested the effect of worry on conditioned responding directly following the manipulation. Future studies could explore whether worry also affects the consolidation of fear memory (e.g., 24 hours later). An alternative explanation for the observed enhancement of fear responding is that worry may have induced anticipatory anxiety. However, induction of an anxious state would probably have resulted into a general increase of startle responding to the context. Given that the manipulation did not yield a difference between the worry and control condition in startle responding to the context (noise alone trials), we do not consider this explanation as very tenable (see Results page 8). Further, as our manipulation incorporated the three characteristics of worrying (i.e., the repetitive nature, negative content, and abstract style of thinking) [6], [12], the exact mechanism of the fear enhancement remains unclear. As such, this study can only be regarded as a first step, and future studies could disentangle the effects of the different components of worrying to explore which components may be responsible for the fear enhancing effects.

Germane to this issue is the lack of indexing the different components of worrying in our manipulation check. However, to our knowledge, no golden standard exists for assessing the separate components of worry. The evaluation of one's own thinking style by self-report (e.g., indicating the degree to which thinking is abstract, verbal or visual) is notoriously unreliable [32]. Moreover, a potential negative side effect of verbalizing thoughts is that it also may influence the process under investigation. Another potential limitation of our experimental design may be our control condition. In the present control condition we utilized a manipulation (i.e., difficult, neutral questions) to exert optimal control over the content of thinking. However, one may argue that control questions could alternatively lead to distraction. The difficulty of designing an appropriate control condition has also been recognized by other researchers in the field: the alternative of a passive control condition (i.e., doing nothing) may allow naturally occurring worry processes to take place [33], whereas any active control condition may act as a distraction [5]. Nevertheless, as fear responses remained stable after our control manipulation, the observed differences between the conditions can probably be attributed to the worry manipulation.

Another related point of concern regarding our manipulation is that a number of participants failed to comply with the manipulations. Depending on the idiosyncratic tendency to worry in daily life, for some individuals it may be difficult to engage in worry upon instruction (i.e., some participants from our worry condition reported having spent more time thinking about other, unrelated things than about the worry questions), whereas other individuals will habitually start to reflect on the aversive experience regardless of the instructions (i.e., some of our control participants spent more time spontaneously recalling the electrical stimulus than answering the control questions). This raises the issue of how worry and control manipulations could be improved. An alternative approach is to capitalize on individual differences in the tendency to worry (e.g., trait worriers). Our finding that participants who failed to engage in worrying were characterized by lower state anxiety scores (excluded participants, see page 7) supports the notion of individual differences in the susceptibility for induction of negative thinking styles. We further suggest that the efficacy of the control induction may be improved by using a (neutral) computer game or a reaction time task that fully occupies the mental activity, minimizing the possibility for unintentional worrying.

In sum, the present results suggest that worry after initial fear acquisition may affect the formation of fear memory and impair fear extinction. This study opens up new avenues to experimentally investigate the effect of cognitive dysfunctional processing styles on associative fear learning by incorporating these processes within a traditional discriminative fear conditioning paradigm.

## Materials and Methods

### Participants

Sixty-nine healthy undergraduate students (73.9% female, age *M* = 22.2 years) participated in the study in return for course credits or a small monetary reward (seven Euros). This study has been approved by the ethical committee of the University of Amsterdam, and written informed consent has been obtained from all participants. All participants were screened to be free from any medical condition that would contraindicate participation: pregnancy, seizure disorder, cardiovascular disease, visual or hearing problems. Participants were randomly assigned to the Worry condition or Control condition with the restriction that groups were matched on sex.

### Manipulation

The experimental condition involved induction of worry after fear acquisition by presenting catastrophic questions regarding the participants' tolerance for the stressor (UCS) (adapted from [6], [34]). Subjects in the Control condition received neutral questions on societal matters. Six questions were sequentially presented on the screen for 15 s for both the Worry and Control condition. Each question was followed by a cue ‘*Think about this question. Try to answer and remember the question as well as possible*.’ for 10 s, and after another 20 s the next question was presented.

#### Worry condition

Questions consisted of the electric stimulus and subjects' reactions to it. Before the first question, participants received the instruction to repeat each question sub-vocally. The following questions (translated from Dutch, see Appendix S1) were presented in random order:

1. *What if there will be more electrical stimuli, will I be able to tolerate them?*
2. *Why exactly have I chosen to participate in a study with electrical stimuli?*
3. *What if I cannot take the electrical stimuli anymore and have to quit the experiment?*
4. *What happens if they discover that my reaction to the electrical stimuli is abnormal in a certain way?*
5. *What if the electrical stimuli in the next phases will be much more painful?*
6. *What happens if the electrical stimuli are somehow bad for me?*

#### Control condition

The six control questions (Appendix S1; adapted from [6]) were demanding, aimed to fully engage the working memory and to maximize control over participants thinking activities during the induction. Participants were asked to solve the questions and remember the answers, thereby enhancing motivation for putting effort in finding answers. An example item was: *How many countries are member of the European Union? Which countries?*

### Apparatus and materials

#### Setup

The experiment was run on a Pentium IV 3 GHz PC. The software program ‘Presentation’ (Version 12.2) managed the display of the CSs and the expectancy rating scale and employed a trigger signal to initiate UCS delivery. It also recorded the expectancy ratings. The software program Vsrrp98 v7.6c (Versatile Stimulus Response Registration Program, 1998; Technical Support Group of the Department of Psychology, University of Amsterdam) managed registration of startle amplitudes and skin conductance. In addition, this program produced 60–70 dB constant background noise.

#### Stimuli

The conditioned stimuli (CS1^+^ and CS2^−^) presented during acquisition and extinction comprised two geometrical figures (a brown circle and a grey square) that were similar in brightness. The stimuli were presented in the middle of a black screen on a 19-inch computer monitor. During acquisition, one of the figures (CS1^+^) was most of the time followed by an UCS, while the other figure (CS2^−^) was never followed by an UCS. Assignment of the slides as CS1 and CS2 was counterbalanced across participants. The unconditioned stimulus (UCS) constituted of a 2-ms electric stimulus produced by a Digitimer DS7A constant current stimulator (Hertfordshire, UK). The UCS was administered to the left wrist via a pair of standard Ag/AgCl electrodes filled with electrolyte gel (Signa, Parker) [35]. UCS intensity was individually set by each participant to the level “difficult to tolerate, but not painful".

### Data Collection

#### Fear-potentiated startle (FPS)

The eyeblink component of the startle response was measured by activity recording of the orbicularis oculi electromyogram (EMG). The acoustic startle probe consists of a 40-ms duration, 104 dB burst of white noise with a near instantaneous rise time, presented binaurally by headphones. Two 7-mm Ag/AgCl electrodes filled with electrolyte gel were positioned approximately 1 cm under the pupil and 1 cm below the lateral canthus. In order to maintain electrically identical paths and reduce common noise, the ground reference was placed ±3 cm below the orbicularis oculi pars orbitalis on an electrically neutral site [19]. The eyeblink EMG activity was measured using a bundled pair of electrode wires connected to a front-end amplifier with an input resistance of 10 MΩ and a bandwidth of DC-1500 Hz. To remove unwanted interference, a notch filter was set at 50 Hz. Integration was handled by a true-RMS converter (contour follower) with a time constant of 25 ms. The integrated EMG signal was sampled at 100 Hz. Startle responses were identified allowing onset between 10–120 ms after probe onset and peak amplitudes were identified from 20 ms after startle onset to 200 ms following this probe.

#### Skin conductance response

Skin conductance was recorded through electrodes attached to the medial phalanges of the second and fourth fingers of the non-preferred hand. SCR elicited by the CS were registered each 0.5 s. The skin conductance responses were calculated by subtracting a baseline of the mean 2 s before CS presentation from the maximum of the following 7 s during CS presentation [19], . Although many previous studies examined the first interval response (FIR) or second interval response (SIR), more recent work suggests that the utility in distinguishing between FIR and SIR is limited and the ‘Entire interval response’ (EIR) scoring method is recommended [36]. The EIR method eliminates the risk that “responses may be underestimated when the response occurs near a previously established boundary between the FIR and SIR or when the latency of the peak response shifts over trials" ([36], p.993).

#### UCS Expectancy ratings

Expectancy of the UCS was rated online during CS presentations on a continuous scale anchored ‘Certainly no electric stimulus’ (*−5*) to ‘Uncertain’ (*0*) to ‘Certainly an electric stimulus’ (*5*). Ratings were registered on a 200 point scale (−100 to 100).

### Subjective assessments

#### STAI-T and STAI-S

Trait anxiety and state anxiety were assessed by Spielberger's State-Trait Anxiety Inventory (Dutch version: [41]). The STAI-T and STAI-S are 20 items self-report questionnaires that measure participants' predispositions to anxiety and state anxiety respectively, and have good psychometric properties [41].

#### UCS Characteristics

At the end of the experiment participants were asked to complete the post experimental UCS Characteristics questionnaire measured on VAS scales (*0–100*) on the (a) (un)pleasantness of the electric stimulus, anchored from ‘Not unpleasant’, to ‘Unpleasant’ to ‘Very unpleasant’ (b) the intensity of UCS, anchored from ‘Light’, to ‘Intense’ to ‘Intolerable’, (c) the degree to which the electric stimulus frightened them, anchored from ‘Not at all’ to ‘Moderately’ to ‘Very strongly’ [42].

#### Manipulation check

The manipulation check questionnaire (adapted from [6], [34]) consisted of eight items, which aimed to retrospectively assess serious participation during the 6 min thinking induction. The following questions were presented: 1) How well one had been able to think about the questions (‘0’ = *Not at all* to ‘4’ = *Very*). 2) What percentage of time had been spent thinking about the questions (0–100%). 3) What percentage of time had been spent thinking about things unrelated to the questions (0–100%). 4) What percentage of time had been spent recalling the electric stimulus (0–100%). 5) During the induction, what percentage of time the participant had been having bodily sensations versus thoughts (0–100%). 6) How distressing it was to think about the questions (‘0’ = *Not at all* to ‘4’ = *Very*). 7) How strongly one had felt obliged to think about the questions (‘0’ = *Not at all* to ‘4’ = *Very*). 8) How well one had found answers on the questions (‘0’ = *Not at all* to ‘4’ = *Very Well*).

### Experimental procedure and design (Figure 1)

After attachment of all electrodes, participants were asked to fill out the State Anxiety Inventory (STAI-S). Next, UCS intensity was individually calibrated. Then, participants were instructed about the conditioning procedure, that is, one of two figures will sometimes be followed by an electric stimulus whilst the other will never be followed by an electric stimulus.

In the *Habituation* phase, eight acoustic startle probes were delivered to reduce initial startle reactivity, allowing discriminative emotional effects on startle reactivity during the experimental procedure [43]. In the *Acquisition* phase, partial reinforcement of the feared stimulus (CS1^+^) was implemented to delay the onset of extinction [44]. CS1^+^ and CS2^−^ were both presented 8 times (CS1^+^ was 6 times followed by the UCS) semi-randomly with the restriction of no more than two consecutive presentations of either CS1^+^ or CS2^−^. Both stimuli were presented for 8 s, the startle probe was delivered 7 s after stimulus onset (late probe), and for CS1^+^ trials the UCS was delivered at 7.5 s. The inter-trial intervals (ITI) varied between 16–29 s with a mean of 22 s, during which startle probes (Noise Alone trials, NA) were delivered. Throughout the first 5 s of every stimulus presentation, participants were required to rate their expectancy of an electric stimulus by shifting a pointer on a bar.

Prior to the *Manipulation*, participants received online instructions that no electrical stimuli or loud noises would be administered during this phase. Then participants were asked to concentrate and think thoroughly about the coming questions. Also, it was noted that the experiment would continue afterwards. For both conditions, the experiment continued with *Extinction*. After 1 NA trial, the unreinforced CS1^−^ (no UCS) and CS2^−^ were presented 12 times randomly with another 8 NA trials. *Reinstatement* was implemented by delivery of one unsignaled UCS. Following another ITI (17 s) and after 1 NA trial, *Relearning of extinction* consisted of 6 presentations of unreinforced CS1^−^ and CS2^−^ semi-randomly with another 5 NA trials. Together, a startle probe was delivered during each CS and each ITI, resulting in a total of 83 probes (habituation: 8, acquisition: 24, extinction: 33; re-extinction: 18).

Afterwards, electrodes were removed and participants completed the post-experimental questions regarding the characteristics of the UCS [42], the Manipulation Check questionnaire, exit-questions, and the STAI-T. Finally, participants from the Worry condition were debriefed, reaffirming the actual safety of the electric stimulus.

### Data reduction and data analysis

For SCR analyses, no significant acquisition effect and no effects of the manipulation were obtained. Therefore, the SCR data are not presented.

#### Participant Exclusion

To ensure the validity of our inductions (see Manipulation and Appendix S1), we implemented a manipulation check (MC; see Manipulation check above and Appendix S2). In total, twenty-one participants (Control; *n* = 11; Worry; *n* = 10) had to be excluded from further analyses because of failure to comply with the instructions. Compliance with the instructions was necessary for the effect to occur, as analyses on the total sample did not reveal significant condition differences, *F*<1.7. Two participants were excluded because they reported not having taken instructions seriously. The other participants were excluded because they indicated 1) that they did not feel inclined to think about the questions (score 0; *n* = 7), 2) to have spent *more or equal time* thinking about things unrelated to the induction questions than about the questions and the electric stimulus in the Worry condition (*n* = 6), or 3) to have spent *more or equal time* thinking about the electric stimulus than about the induction questions in the Control condition (*n* = 8). The final sample consisted of 48 participants: Worry (n = 23) and Control condition (n = 25).

#### Data reduction

For FPS analyses, six additional participants were excluded because of technical problems (e.g., noise in the EMG signal, EMG responses exceeding the measurement scale) (*n* = 6) and one participant only lacked FPS data of the re-extinction phase due to a problem with the electrode attachment (*n* = 1). Taken together, startle analyses are based on the data of 42 participants (Worry *n* = 20, 3 male; Control *n* = 22, 7 male), with reinstatement analyses and re-extinction on 41 participants. UCS expectancy analyses are based on the complete data of 48 participants (Worry *n* = 23, 4 male; Control *n* = 25, 8 male), with reinstatement and re-extinction analyses on 47 participants. The FPS and UCS expectancy samples did not differ in terms of age, reported trait and state anxiety, UCS intensity and UCS evaluation (*t*s(40)<1.5). Further note that analyses of UCS expectancy over the FPS subset (n = 42) revealed a similar pattern of results as analyses over the entire sample. In specific, analyses did also not reveal any differences between conditions at immediate testing (post manipulation; *F*s(1,40)<1.3), and similar results were observed at reinstatement testing and re-extinction (CS-Type×Condition; *p*s<.057), except that the difference between conditions during extinction no longer reached significance (CS-Type×Trial×Condition; *F*(1,40)<2.5).

#### Missing values

Startle measurements that showed recording artifacts or excessive baseline activity were discarded by the Vs.rrp98 v7.6c software program, resulting in 2 out of the 3132 discarded startle measurements. Outliers (>3 SD from the mean) within participants were removed (yielding the top 26 trials). In addition, outliers between participants were removed, calculated from the mean over participants separately per condition (yielding 2 excluded trials) [45]. The resulting total missing data (i.e., 30 of 3132 trials) were replaced with the mean of the valid response before and/or after that data point within each participant (0–4 per participant). To account for individual differences in startle response magnitudes, blink data were subject-wise z transformed (based on all startle responses during acquisition, extinction and re-extinction) [46]. These z-scores were next converted to T-scores (T = (z * 10)+50) in order to obtain unidirectional values [47]. Because startle magnitudes vary strongly, the factor Trial was based on the average of two successive trials and UCS expectancy ratings were averaged similarly.

#### Data analysis

To check for between condition differences, the STAI-T, Manipulation Check and UCS characteristic questionnaires were analyzed using independent *t*-tests. FPS and UCS expectancy data were analyzed with repeated measures ANOVAs with condition (Worry vs. Control) as between-subjects factor and CS-Type (CS1 vs. CS2) and Trial as within-subjects factors. To test the major hypotheses, planned contrasts were performed. Follow-up analyses were performed following significant ANOVAs by pairwise comparisons or separate within-condition ANOVAs.

*Acquisition* was analyzed by comparing the differential response (CS1 vs. CS2) at the start (trial 1, 2) of acquisition to the end (trial 7, 8) of acquisition. To analyze the immediate effect of the *Manipulation*, the differential response (CS1 vs. CS2) at the end of acquisition was compared with the start (trial 1, 2) of extinction. To test for magnitude of *Extinction*, differential responding (CS1 vs. CS2) at the start of extinction was compared with the end (trial 11, 12) of extinction. The *Reinstatement* effect was assessed by comparing the differential responding (CS1 vs. CS2) at the end of extinction with the first test trials (trial 1, 2; start of re-extinction). *Relearning of extinction* was tested identical to extinction. We performed separate additional analyses for the startle responses during the ITIs in order to control for non-specific differences in arousal between the two conditions, with Condition as between-subject factor and Trial (NA trials) as within-subject factor. A Greenhouse-Geisser (GGε) procedure was applied in case of violation of the sphericity assumption. An alpha level of .05 was used for all statistical analyses. False Discovery Rate (FDR) correction [48] was applied to all post-hoc comparisons when indicated. Partial eta squared (ηp2; [49]) was used as index of effect size. For experimental studies an effect size of *η*_p_^2^ = .01 is considered small, *η*_p_^2^ = .09 medium, and *η*_p_^2^ = .25 large [45], [49].

## Supporting Information

Appendix S1 **Manipulation.** Dutch version of Worry questions and Control questions in both Dutch and English.
(DOC)

Appendix S2 **Manipulation Check.** Results of the Manipulation Check.
(DOC)
