# Supplementary material for: Worrying Affects Associative Fear Learning: A Startle Fear Conditioning Study
Source: PLoS One. 2012 Apr 13;7(4):e34882. doi: 10.1371/journal.pone.0034882 (PMC3325932; doi:10.1371/journal.pone.0034882)
Supplement: Appendix S1 — Manipulation. Dutch version of Worry questions and Control questions in both Dutch and English. (DOC) [file pone.0034882.s001.doc]

**Appendix S1. Manipulation**

Dutch version of Worry questions and Control questions in both Dutch and English.

Cue: "Denk over de vraag na. Probeer de vraag zo goed mogelijk te beantwoorden en te onthouden".

**Worry condition**

1. "Wat nou als er nog meer prikkels volgen, zou ik ze dan kunnen verdragen?"
2. "Waarom heb ik nou voor dit onderzoek met die elektrische prikkels gekozen?"
3. "Wat als ik de prikkels niet meer aankan, en ik met het experiment moet stoppen?"
4. "Wat als ze er achter komen dat mijn reactie op de prikkel - op wat voor manier dan ook - niet normaal is?"
5. "Wat nou als de prikkels in de volgende fasen veel pijnlijker voelen?"
6. "Wat nou als de prikkels toch slecht voor mij blijken te zijn?"

**Control condition**

1. "Hoeveel landen heeft de Europese Unie? En welke landen zijn dit?"

*How many countries are part of the European Union? Which countries are they?*

1. "Hoeveel dagen heeft een schrikkeljaar? En hoe vaak komt zo'n schrikkeljaar voor? Hoeveel dagen zitten er dan in 8 jaar? "

*How many days does an intercalary year have? And how often does such a year occur? Then, how many days are there in eight years?*

1. "Hoeveel faculteiten heeft de Universiteit van Amsterdam? Hoe heten deze faculteiten?"

*How many faculties does the University of Amsterdam have? What are these faculties called?*

1. "In hoeveel landen praat men Engels? Welke landen zijn dit?"

*In how many countries do people speak English? In which countries exactly?*

1. "Hoeveel buurten kent Amsterdam? Welke buurten zijn dit?"

*How many districts does Amsterdam have? Which districts exactly?*

1. "Uit hoeveel staten bestaat de Verenigde Staten? Probeer 20 staten te onthouden."

*How many states do the United States have? Try to remember twenty states.*
