# Supplementary material for: Worrying Affects Associative Fear Learning: A Startle Fear Conditioning Study
Source: PLoS One. 2012 Apr 13;7(4):e34882. doi: 10.1371/journal.pone.0034882 (PMC3325932; doi:10.1371/journal.pone.0034882)
Supplement: Appendix S2 — Manipulation Check. Results of the Manipulation Check. (DOC) [file pone.0034882.s002.doc]

**Appendix S2. Manipulation Check**

Participants indicated to have been able to think about the questions presented on the computer screen (Worry: *M* = 3.00, *SD* = .90; Con: *M* = 2.80, *SD* = 1.00), and also reported that they felt obliged to persistently think about the questions (Worry: *M* = 2.04, *SD* = .82; Con: *M* = 2.44, *SD* = 1.00). Furthermore, participants indicated that they had spent on average 70% of the time thinking about the questions that were presented (Worry: *M* = 66.74, *SD* = 14.59; Con: *M* = 70.80, *SD* = 18.69). They also reported that it was somewhat distressing to think about the questions (Worry: *M* = .96, *SD* = .93; Con: *M* = 1.44, *SD* =1.00). Groups did not significantly differ on these variables, all *t*s < 1.8.

As expected, the groups differed on a number of additional items of the Manipulation Check Questionnaire: Participants in the Worry condition reported to have thought significantly more about the electric stimulus during the induction than the Control group (Worry: *M* = 39.57, *SD* = 26.54; Con: *M* = 13.20, *SD* =13.06), *t*(46) = 4.31, *p*< .0001. In addition, participants in the Control condition indicated that they had more difficulty finding answers to the questions compared to the Worry condition (Worry: *M* = 2.91, *SD* = 1.12; Con: *M* = 1.84, *SD* = .95), *t*(46) = 3.57, *p* = .001. Probably, this latter difference was due to the nature of the control questions, as these questions were hardly possible to answer correctly within time.
